# Optimisation of self-healing of bio-foamed concrete bricks pores using *Bacillus tequilensis* under different temperature and CO_2_ curing conditions

**DOI:** 10.1038/s41598-022-05659-0

**Published:** 2022-02-17

**Authors:** Abdullah F. Alshalif, M. Irwan Juki, Husnul Azan Tajarudin, N. Othman, Adel Ali Al-Gheethi, S. Shamsudin, Wahid Altowayti, Saddam Abo Sabah

**Affiliations:** 1 Jamilus Research Centre for Sustainable Construction (JRC), Faculty of Civil and Environmental Engineering, Universiti Tun Hussein Onn Malaysia, 86400 Parit Raja, Johor Malaysia; 2 Division of Bioprocess, School of Industrial Technology, Universiti Sains Malaysia, 11800 Pulau Pinang, Malaysia; 3 Micro-Pollutant Research Centre (MPRC), Faculty of Civil and Environmental Engineering, Universiti Tun Hussein Onn Malaysia, 86400 Parit Raja, Johor Malaysia; 4 Sustainable Manufacturing and Recycling Technology, Advanced Manufacturing and Materials Center (SMART-AMMC), Universiti Tun Hussein Onn Malaysia, 86400 Parit Raja, Johor Malaysia

**Keywords:** Civil engineering, Microbiology, Climate sciences, Environmental sciences, Engineering

## Abstract

The self-healing of bio-concrete cracks and pores have been utilised worldwide to improve the properties of bio-concrete using different types of bacteria. Meanwhile, no published research was conducted to heal bio-foamed concrete bricks (B-FCB) pores using *Bacillus tequilensis.* Previous studies focused on the concentration of bacteria and neglect other factors that could affect the healing process. This research aimed to optimise the healing ratio of B-FCB pores using four factors: *B. tequilensis* concentration, concrete density, temperature and CO_2_ concentration. Initial water absorption (IWA) and water absorption (WA) were used as responses in statistical methods, namely, factorial and response surface methodology (RSM). *B. tequilensis* species was isolated from cement kiln dust, produced in a powder form, then subjected to simulate test using a special medium consisting of foamed concrete materials to check the survival ability in B-FCB. SEM, EDX, and XRD were used to investigate the healing process of B-FCB pores. The results revealed that the decrement ratios of IWA and WA of B-FCB were 52.8% and 29.1% compared to FCB, respectively. SEM results reflect the healing that occurred in B-FCB pores, mostly healed via precipitation of CaCO_3_ as demonstrated on the XRD results.

## Introduction

Self-healing of concrete cracks and pores by precipitation of calcium carbonate (CaCO_3_) on bacteria surfaces has been regarded as a sustainable solution and environmental-friendly for a wide range of bio-concrete applications^1,2^. For instance, in Europe, around 50% of the annual construction budget is spent on maintenance which increases the cost of repairing reinforced concrete due to the micro-cracks which developed into serious cracks^3^. About 27% of highway bridges have corrosion deterioration in the USA, requiring replacement or repair. The deterioration in concrete may be due to the age of concrete, especially on old constructions built decades ago. However, the main reason for concrete deterioration is the high permeability of the concrete that allows water and other aggressive elements to enter^4,5^.

Self-healing technology in concrete is considered a beneficial method to reduce the maintenance cost on a long life span of concrete^6^. Recently, self-healing technology is being used in bio-concrete with bacteria that could produce carbonic anhydrase and urease enzymes^7–9^. The bacteria may reduce atmospheric CO_2_ concentration by sequestering it into bio-concrete in the form of calcite CaCO_3_. However, this direction is not suitable for reinforced concrete because a carbonation attack may occur due to CO_2_ sequestration resulting in steel corrosions^10^. Sequestration of CO_2_ into bio-concrete can be accelerated via bacterial enzymes reaction such as carbonic anhydrase enzyme and natural carbonation of the concrete^7,8^. Therefore, bio-sequestration of CO_2_ can be more useful in non-reinforce concrete such as bricks. Consequently, the applications of self-healing technology in bio-concrete are increasing continuously worldwide due to economic, environmental and sustainability aspects^11,12^. These reasons have made many researchers conducted studies to improve self-healing in bio-concrete^13–15^. However, the correlation between the internal and external factors such as density of concrete, bacteria concentration, curing temperature, and CO_2_ concentration that affects self-healing processes in bio-concrete has received little attention. Also, there are no publications on the optimisation of self-healing on bio-concrete.

Statistical optimisation methods such as factorial and RSM may help identify the effect of internal and external factors that may affect the self-healing process in bio-concrete. A few studies in this field used optimisation, such as the optimum medium that promoted the growth of *Lysinibacillus boronitolerans* YS11 was identified using the screening process of Plackett–Burman design; however, the internal factors considered with neglected to external factors^16^. Other factors that affect self-healing and microbial-induced CaCO_3_ precipitation (MICP) include the concentration of soluble calcium, the concentration of carbonate, pH, and availability of nucleation sites to form calcium carbonate crystal^17^. Therefore, it is difficult to optimise self-healing on bio-concrete using internal factors only without considering external factors vice versa.

This research aims to optimise self-healing of bio-foamed concrete bricks (B-FCB) pores with consideration of internal factors, namely density of concrete and *Bacillus tequilensis* concentration, and external factors, namely temperature and CO_2_ concentration.

## Materials and methodology

Producing foamed concrete bricks (FCB) and bio-foamed concrete (B-FCB) consist of several steps: the preparation of materials and specimens, curing, statistical analysis (factorial and RSM) and responses description as explained in detail in the following subsections.

### Preparation of FCB and B-FCB materials

Ordinary Portland cement, sand, water, synthetic foaming agents were the materials used to produce FCB. *B. tequilensis* was added to the same materials to produce B-FCB. The cement was manufactured by Malaysia Berhad (CIMA), located in Bahau, Negeri Sembilan, with the composition and specification of OPC complying with all requirements were defined by BS 197-1:2000. The sand was sieved using a sieved machine with 1 mm mesh size^18^, according to BS 882-1992. A water pipe was used for a foamed concrete mix of FCB and B-FCB. The same type of water was used to diluted synthetic foaming agent with a ratio of 1:20 and then aerated to a density of 65 kg/m^3^ according to ASTM C796 Standard test method for foaming agents to be used in producing cellular concrete using preformed foam^19,20^. The *B. tequilensis* was isolated, subjected to several assays and produced in powder form to be ready to add in bio-foamed concrete water^21^. *B. tequilensis* was used as one of the main factors in this study, in 2^k^ factorial design and RSM design with the following range of concentration (3 × 10^5^ to 3 × 10^7^) cell/mL. However, the concentration values cannot be written in the Minitab software as following format (3 × 10^5^ to 3 × 10^7^) cell/mL. Therefore, 5, 6 and 7 were used to represent the power of *B. tequilensis* concentration in both designs.

### Specimens preparation and curing process

The foamed concretes, with and without *B. tequilensis*, were prepared by first placing an amount of cement and fine sand into a mixer machine. Then, water was added into the mixer. A foaming agent was used to balance the target fresh density. Up to this step, the process was to produce FCB.

**Figure 1:**
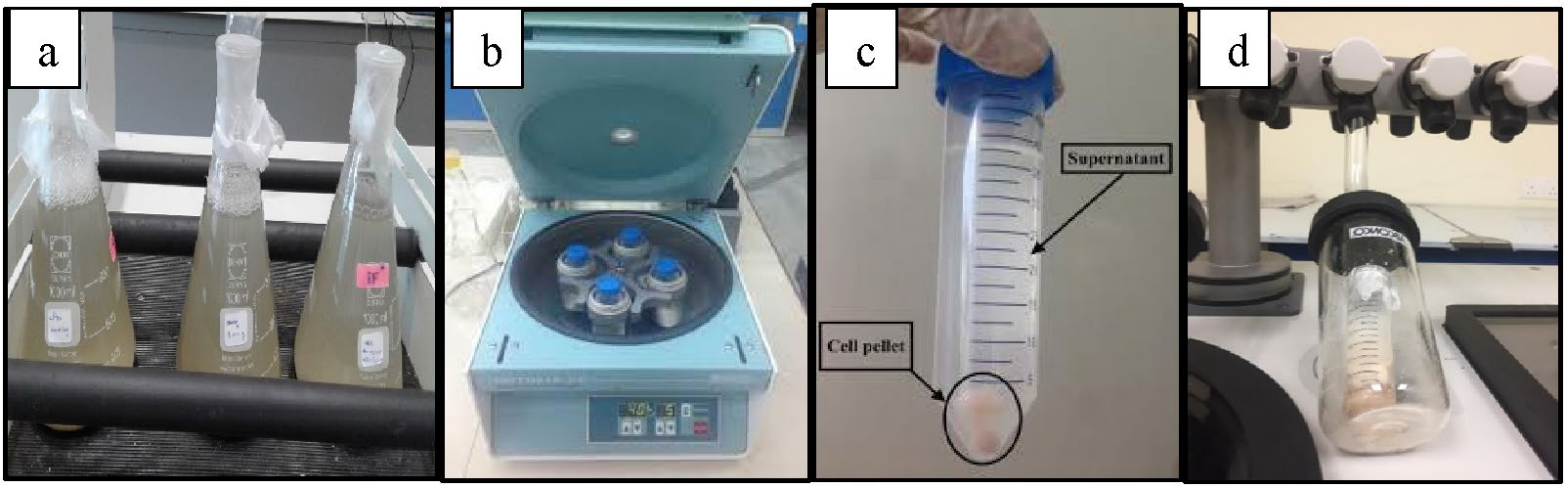
Process of producing *B. tequilensis* into a powder form: (**a**) the culture being shaken, (**b**) centrifugation process, (**c**) the separated cells pellet and medium supernatant, (**d**) freeze-drying of the cell pellets.

Bio-foamed concrete was produced by adding *B. tequilensis* into water five minutes before adding the water to produce B-FCB. Before that, *B. tequilensis* was isolated from cement kiln dust to acclimatise in the bio-concrete environment. *B. tequilensis* cells were first sub-cultured in a new broth medium at 30 °C and shaken mechanically during the incubation time for 24 h as shown in Fig. 1a. Then, the cells were immediately harvested by centrifugation at 4000 rpm for 5 min at normal temperature, as shown in Fig. 1b. The centrifugation will separate the cells (as cells pellet at the bottom of the tube) and the medium (as supernatant), as demonstrated in Fig. 1c. Bacteria cells pellet were collected in an autoclaved bottle and put into a freeze- dryer at − 40 °C under pressure less than 0.133 mbar with no heat or energy supplied during the process for 96 h, as shown in Fig. 1d.

Three specimens of foamed concrete bricks with the size of (215 × 100 × 65 mm) without *B. tequilensis* were prepared as control, and three with *B. tequilensis* at the same size for each response for initial water absorption (IWA) and water absorption (WA) in each run. The specimens were taken out from the moulds after 24 h of demoulding in the atmosphere.

**Figure 2:**
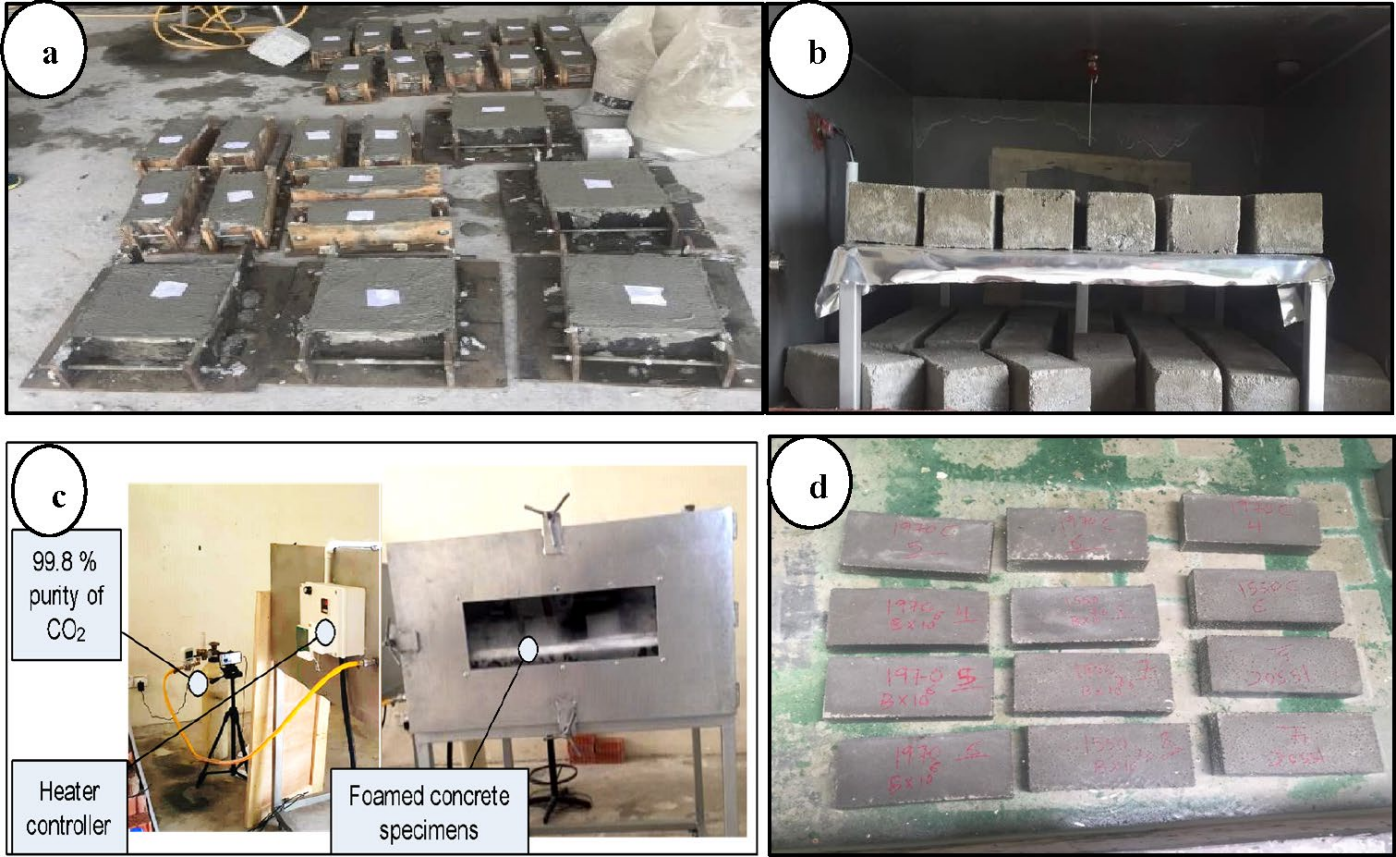
Concrete specimens (**a**) demoulding specimens, (**b**) placing specimens in a chamber, (**c**) curing of specimens in chamber (**d**) curing of specimens in water.

All specimens were placed in a chamber for 72 h at 50 °C to make sure the specimens are dry before starting the curing process, as shown in Fig. 2. The curing conditions in the chamber were different from one run to another according to the suggested conditions by 2^k^ factorial and RSM analysis throughout 28 days. However, the specimens of the WA test were emerged in water for 24 h after the curing period in the chamber, as shown in Fig. 2d.

### Statistical analysis methods

The design of experiment (DOE) for 2^k^ factorial and RSM was done using Minitab 18. The 2^k^ factorial design is the most common technique used in DOE, which is helpful in screening important factors of the experiment. With time and fund constraints throughout the entire study, the 2^k^ factorial design was given priority. The effect of *B. tequilensis* on self-healing of B-FCB was investigated together with the following factors: density (D), temperature (T), *B. tequilensis* concentration (B), and CO_2_ concentration (CO_2_), then compare to the control sample FCB under the same conditions for each run.

The design scheme is listed in Table 1. Only a single run was implemented in each corner, and 3 centre points with a total of 11 runs were involved. The responses for this study were IWA and WA. Analysis of variance (ANOVA) was applied to rank the main effects and to analyse the interactions among the input parameters. The ANOVA results can detected by P-value whether the curvature is significant or unsignificant. Therefore, the use of factorial method can conclude that curvature is significant to use RSM such as a central composite design to analyze the data to build an equation of the model. Therefore, the DOE results can suggest an insight into further experimental direction for process optimisation. Therefore, 8 runs plus 2 centre runs were suggested by RSM based on the curvature value as presented in ANOVA results at the screening stage as shown in Table 1. Therefore, 21 runs were conducted to optimise IWA and WA, as shown in Table 1. ANOVA is essential in 2^k^ design to come up with a structured analysis of results. It studies the effectiveness of the analysis with five major steps:

- Calculating the sum of squares (*SS*).
- Determining the degrees of freedom (*DOF*).
- Calculating the mean squares (*MS*).
- Determining the test ratio *F*_*0*_.
- Drawing conclusions from the results.

**Table 1:** The mixture design and factor levels in each run according to 2^k^ factorial and RSM design.

Run no	Density (kg/m^3^)	Cemnet (kg/m^3^)	Fine sand (kg/m^3^)	Water (L/m^3^)	°C	CO_2_ (%)	B (cell/mL)
**Runs of 2**^**k**^** factorial design**							
1	1300	553.2	746.8	276.6	27	10	3 × 10^5^
2	1800	766.0	1034.0	383.0	27	20	3 × 10^5^
3	1300	553.2	746.8	276.6	27	20	3 × 10^7^
4	1800	766.0	1034.0	383.0	27	10	3 × 10^7^
5	1300	553.2	746.8	276.6	40	20	3 × 10^5^
6	1800	766.0	1034.0	383.0	40	10	3 × 10^5^
7	1300	553.2	746.8	276.6	40	10	3 × 10^7^
8	1800	766.0	1034.0	383.0	40	20	3 × 10^7^
9	1550	659.5	890.4	329.7	33.5	15	3 × 10^6^
10	1550	659.5	890.4	329.7	33.5	15	3 × 10^6^
11	1550	659.5	890.4	329.7	33.5	15	3 × 10^6^
**Runs of continues RSM design**							
12	1550	659.5	890.4	329.7	33.5	10	3 × 10^6^
13	1550	659.5	890.4	329.7	33.5	20	3 × 10^6^
14	1550	659.5	890.4	329.7	33.5	15	3 × 10^5^
15	1550	659.5	890.4	329.7	33.5	15	3 × 10^7^
16	1550	659.5	890.4	329.7	27.0	15	3 × 10^6^
17	1550	659.5	890.4	329.7	40.0	15	3 × 10^6^
18	1300	553.2	746.8	276.6	33.5	15	3 × 10^6^
19	1800	766.0	1034.0	383.0	33.5	15	3 × 10^6^
20	1550	659.5	890.4	329.7	33.5	15	3 × 10^6^
21	1550	659.5	890.4	329.7	33.5	15	3 × 10^6^

*°C* temperature, *CO*_*2*_ concentration of CO_2_, *B* *B. tequilensis* concentration.

### Responses tests

#### Initial water absorption (IWA) and water absorption (WA)

**Figure 3:**
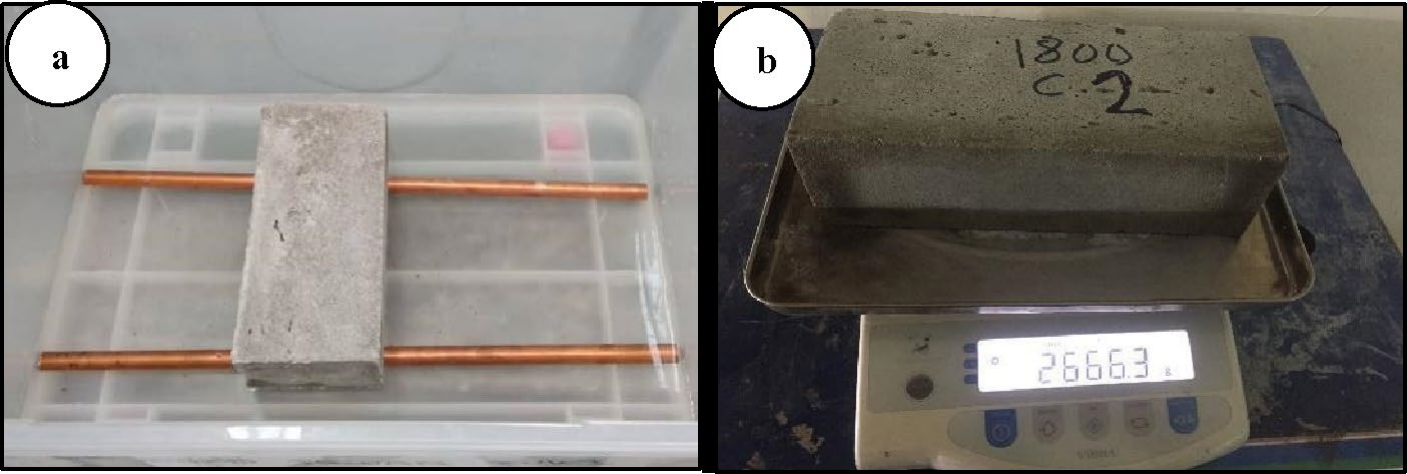
The initial rate of water absorption test. (**a**) Initial water absorption in 1 min. (**b**) The weight of wet brick.

Initial water absorption is one of the parameters conducted in this study to measure the initial rate of water absorbed in FCB and B-FCB according to BS 3921:1985, which can be used as an indicator of the self-healing process occurring on a specimen's surface. The specimens of FCB and B-FCB were cured for 28 days in the chamber according to the suggested conditions by 2^k^ factorial and RSM designs presented in Table 1. However, before curing time, the specimens were placed in the chamber for three days at 50℃ to make sure the specimens were dried. Then, at 28 days of curing, the mass of each specimen was recorded as M_1_. After that, a container of water was placed on a table level. Two steel bars or rods were placed approximately 100 mm far from each other at the bottom of the container with about 4 mm water level. The pre-weighted specimens were placed on the bar, and the water level was observed closely using a measuring gauge to ensure the depth of immersion is maintained at 3 ± 1 mm for 1 min (Fig. 3). After 1 min, the brick specimen was removed from the container and any excess water was wiped off using a dry cloth. The brick specimen was then reweighted and the mass, M_2_, was recorded. The initial water absorption was calculated using the following Eq. (1).

1 $${\text{I}} = \frac{{1000({M_2} - {M_1})}}{A} $$

where I = Initial water absorption (kg/m^2^ min), M_1_ = Weight of dry brick (g), M_2_ = Weight of wet brick (g), A = Net area of the contact surface of brick with water (mm^2^).

After measuring IWA, the specimens of FCB and B-FCB were weighted and recorded as M1. Then, the specimens were submerged completely in two clean water containers, separately under room temperature of 27 ± 2 °C, for 24 h to measure the water absorption. The excess water was wiped off using a dry towel before measuring the weight of the submerged bricks (M_2_). After that, the percentage of water absorption was calculated using the following Eq. (2).

2 $$W = \;\frac{{{M_2} - {M_1}}}{{M_1}} \times \;100 $$

where W = Water absorption in (%), M_1_ = Weight of dry brick (g), M_2_ = Weight of wet brick (g).

### Microstructure analysis

Several tests were used to study microstructure of FCB and B-FCB namely; scanning microscopy morphology (SEM), energy dispersive x-ray (EDX) and X-ray diffraction (XRD). A fragments of concrete were taken after conducted compressive strength test to use for SEM and EDX, which were coated around four minutes before scanning analysis. EDX analysis of concrete samples was conducted to spotted out the weight in percentage of Ca, C and O, which are the main elements to precipitate CaCO_3_^22^. The crystallinity was analysed with XRD to identify the bio-product. Concrete specimen was grinded to be powder form and sieve on the plate with size 300 micron and placed in the plates for XRD to scan with same angle 2*θ*.

## Results and discussion

### Initial water absorption of FCB and B-FCB

**Figure 4:**
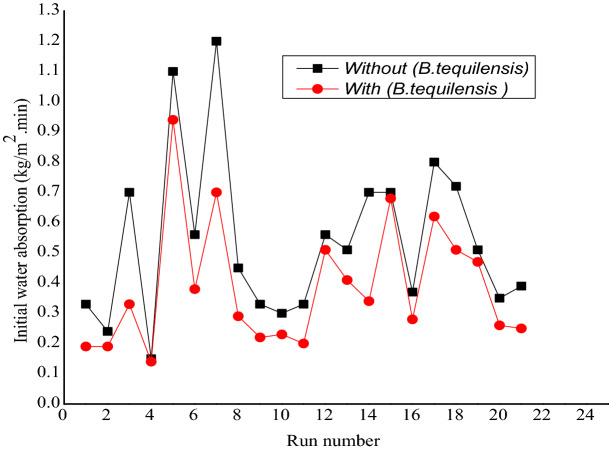
The initial water absorption of FCB and B-FCB.

The results demonstrated the difference of IWA between FCB and B-FCB, which symbolise the initial absorption test rate to measure how quickly the water is being absorbed through the bricks in 1 min. Figure 4 reveals the results of IWA for both types of specimens after 28 days. In general, the IWA was high in FCB specimens compared to B-FCB specimens. The reduction that occurred on IWA of B-FCB was due to the self-healing of the pores on the surface of B-FCB specimens. This is due to the combination of natural sequestration of CO_2_ and biological sequestration via *B. tequilensis* enzymes, enhancing the precipitation of CaCO_3_. Carbonic anhydrase enzyme had been reported as the assistance of CO_2_ fixation^23^. Therefore, the presence of enzymes in B-FCB with high amounts of CO_2_ in the chamber during the curing process caused an increase of CaCO_3_ precipitation, which accelerates self-healing in B-FCB.

Based on Fig. 4, the highest decrements of IWA on B-FCB compared to FCB were at runs 3, 14 and 1 by 52.8%, 51.4% and 42.4%, respectively. The three runs had different levels of each factor, which reflect the significant roles of the factors used on IWA results of FCB and B-FCB. Overall, the decrement of IWA between FCB and B-FCB with runs having a high-density level of 1800 kg/m^3^ was lower than the runs with low and centre levels of 1300 kg/m^3^ and 1550 kg/m^3^, respectively.

The high level of self-healing on the surface of B-FCB for runs 1 and 3 could be due to the high level of porosity when the D was at a low level of 1300 kg/m^3^. In addition, both runs had the same conditions except for *B. tequilensis* concentration, which was 3 × 10^5^ cell/mL and 3 × 10^7^ cell/mL for runs 1 and 3, respectively. Due to that, the highest decrease of IWA occurred at run 3 with a high concentration of *B. tequilensis* compared to run 1. Therefore, the self-healing of the porosity in B-FCB at run 3 was higher compared to run 1. On the other hand, the use of *B. tequilensis* at high concentration also caused a high decrement on IWA with 35.5% at a D level of 1800 kg/m^3^ on B-FCB compared to FCB when the other factors were at the highest levels two as presented in run 8. It was also observed that the IWA increases when the T of curing increases for both FCB and B-FCB as in runs 5 and 7, as shown in Fig. 4.

Even though the IWA was high in runs 5 and 7, *B. tequilensis* in B-FCB has reduced water absorption by 14.5% and 32.1% compared to FCB, respectively. Accordingly, the reduction in run 7 of IWA was almost double compared to run 5 because the concentration of *B. tequilensis* of this run was at a high level compared to the low level of run 7. From the results and discussion above, a strong relationship between *B. tequilensis* concentration and other factors with IWA in FCB and B-FCB and the self-healing of the pores via CaCO_3_ precipitation was observed.

### Water absorption in FCB and B-FCB

The results of WA are almost parallel with IWA. However, WA results are more accurate than IWA because the curing period was 24 h in water, which could be sufficient to indicate the capability of *B. tequilensis* to cause self-healing in B-FCB specimens and the amount of water absorbed. In addition, the IWA was used to indicate the healing process that occurred on the surface of B-FCB compared to FCB, while the WA demonstrated the difference of the healing process on the whole specimen^24^. The test of WA is considered one of the important parameters in bio-concrete technology, indicating the capability of bacteria to heal the pores^25^.

**Figure 5:**
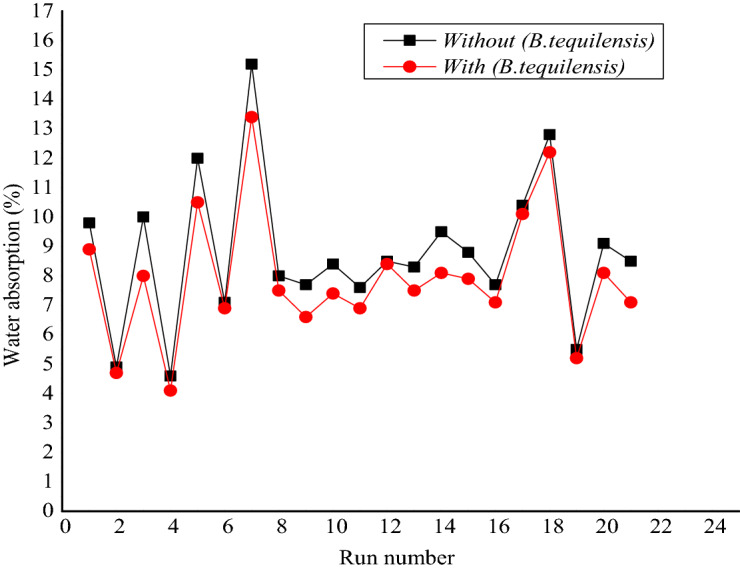
Water absorption results of FCB and B-FCB.

Figure 5 shows the results of WA of FCB and B-FCB, which immersed in water for 24 h after 28 days of curing in the chamber. The results showed that all factors played a vital role in increasing or decreasing WA in the specimens with and without *B. tequilensis*. However, the specimens of B-FCB had a low ratio of WA in all runs compared to FCB. The top three runs with the highest decrease of WA for B-FCB compared to FCB were 5, 3 and 21 giving 29.1%, 20.0%, and 16.4%, respectively. Runs 5 and 3 had a low density level of 1300 kg/m^3^ and run 21 was the centre point with 1550 kg/m^3^. While the maximum decrease of WA of B-FCB compared to FBC with the high level of density was 10.6%. Consequently, the self-healing occurred in all specimens of B-FCB. However, it was at a higher level when the D was low level. The reason could be due to the level of porosity in B-FCB with a low D level, which gives *B. tequilensis* free space to precipitate a high amount of CaCO_3_ compared to B-FCB with a high level of D. On the other hand, the highest WA of both FCB and B-FCB occurred at run 7 when *B. tequilensis* and T were at a high level while D and CO_2_ at a low level. However, WA in B-FCB was lower than FCB by 11.8% because *B. tequilensis* was at a high level in this run.

D is not the only reason that caused the increment of WA. T and CO_2_ could be considered significant factors responsible for decreasing water absorption in B-FCB within the curing time. In addition, *B. tequilensis* concentration, considered the main factor, was causing the reduction of WA on B-FCB. This finding is similar to the previous study reported by Hosseini Balam^25^, who confirmed that bacteria having urease enzyme reacts with the available Ca in cement and CO_2_ that ingress into concrete due to the carbonation process, forming CaCO_3_ on the surface of bacteria which in turn cause remediation of bio-concrete pores and cracks.

It can be summarised that D, T, CO_2_, and *B. tequilensis* play important roles in self-healing and precipitation of CaCO_3_ in B-FCB pores. However, D of B-FCB is considered the key factor in increasing or decreasing WA due to the self-healing process.

### Statistical analysis of both IWA and WA

A total of 11 runs were carried out according to the full 2^k^ factorial design with three centre points. For one replicate design, no internal estimate of error can be calculated. Therefore, all high-order interactions were neglected. Then, the mean squares of the omitted factors are combined to estimate the error. Realising the obvious risk of fitting the model to nuisance factors when conducting the experiment with only one run at each test combination, the variability tests of responses were conducted on the centre runs^26^. The results demonstrated that the standard deviation of IWA and WA were 0.01527 and 0.3000, and the resulting R^2^ was 99.93% and 99.77%, respectively. The small value of standard divisions and the high percentage of R^2^ reflects the highly significant and acceptable results of both IWA and WA.

### Factorial method

#### Significant factors affect the responses

The results of ANOVA for 2^k^ factorial analysis in Table 2 are demonstrating that all factors significantly influenced the IWA with P < 0.05, while two factors, namely *B. tequilensis* and CO_2,_ were insignificant for WA as a single factor with P > 0.05. The initial results indicated that the self-healing process of the pores on the B-FCB surface occurred via acceleration of CaCO_3_ precipitation as a result of the combination reactions between CO_2_ and *B. tequilensis* enzymes. Therefore, CO_2_ and *B. tequilensis* had a significant effect at IWA. On the other hand, CO_2_ and *B. tequilensis* were insignificant to WA because the CO_2_ takes time to penetrate deeply inside concrete resulting in a decrease in bio-reaction, self-healing process, and CaCO_3_ precipitation.

**Table 2:** ANOVA factorial analysis for the effect of factors and their interactions to the responses.

Source	P-value (IWA)	Effect (IWA)	P-value (WA)	Effect (WA)
Model	0.003	–	0.007	–
Linear	0.002	–	0.004	–
Density	0.001	− 0.290	0.001	− 4.675
Bacteria	0.031	− 0.060	0.333	0.225
Temperature	0.001	0.365	0.002	3.625
CO_2_	0.016	0.085	0.429	− 0.175
2-way interactions	0.007	–	0.067	–
Density*bacteria	0.452	− 0.010	0.901	− 0.025
Density*temperature	0.003	− 0.195	0.072	− 0.625
CO_2_*density	0.003	0.105	0.032	0.975
Curvature	0.003	–	0.015	–

In contrast, interactions of the two factors, namely Density*Bacteria, have insignificant P-values for both responses, while the interactions between Density*Temperature were significant for IWA and insignificant for WA with P-values of 0.003 and 0.072, respectively. In addition, the interactions between CO_2_*Density was significant for both responses with P-values of 0.003 and 0.032, respectively. The ANOVA results of factorial analysis summarised that all factors used are important. However, 2^k^ factorial analysis is not enough to optimise the IWA, WA, and self-healing process because the curvature was significant with P-value ˂ 0.05 for both responses, as shown in Table 2. Due to that all factors remain for RSM analysis using central composite design to build an equation of the model.

**Figure 6:**
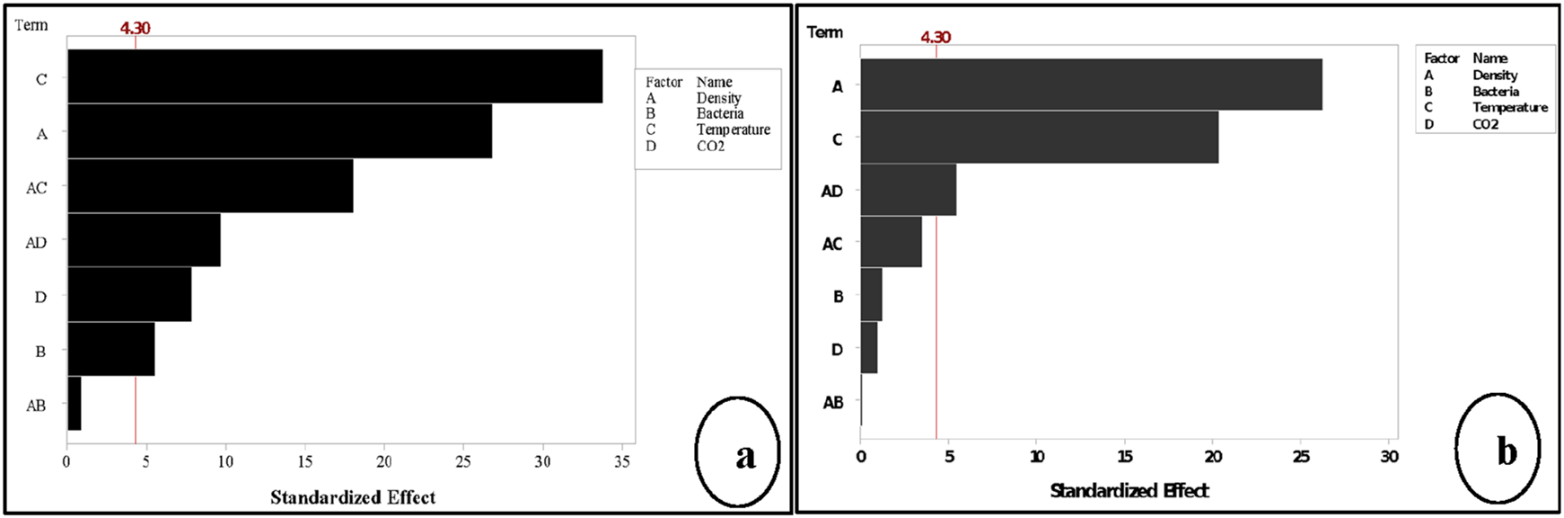
Pareto chart of the standardised effects by the factorial analysis α = 0.05 (**a**) response is initial water absorption (**b**) response is water absorption.

The results indicated that the significant factors of IWA and WA were not the same, as shown in Fig. 6. The highest significant factors on IWA were temperature and density, followed by the interaction between density*temperature and density*CO_2_. CO_2_ and bacteria were also significant in the IWA response. The top significant factors on WA were density and temperature, followed by the interaction between density*CO_2_. The difference in the results between the two responses can be seen clearly in the main factors and their interactions, as shown in Fig. 6a,b. This finding can give strong evidence on the ability of *B. tequilensis* to accelerate sequestration of CO_2_ from the atmosphere into B-FCB pores in the form of CaCO_3_, which in role improves the self-healing process in B-FCB^12,27^. On the other hand, the interaction between density*CO_2_ was significant in both responses, confirming the theory of the previous studies that density and CO_2_ have a strong relationship^27^. Whereas the lower density has the higher CO_2_ sequestration and the higher density has the lower CO_2_ sequestration, which results in precipitation of CaCO_3_ and heals the pores of the concrete^28^. Therefore, the healing of the pores on the surface of the specimens was higher compared to the pores inside the specimens due to the high precipitation of CaCO_3_. CaCO_3_ precipitates more with availability of CO_2_ through natural carbonation process, which was also accelerated via carbonic anhydrase and urease enzymes produced by *B. tequilensis*^29,30^. Therefore, *B. tequilensis* plays an important role on the surface of B-FCB by accelerating the precipitation of CaCO_3_ due to the high concentration of CO_2_ on the surface of the specimens inside the chamber.

#### The performance of the main factors and their interactions

**Figure 7:**
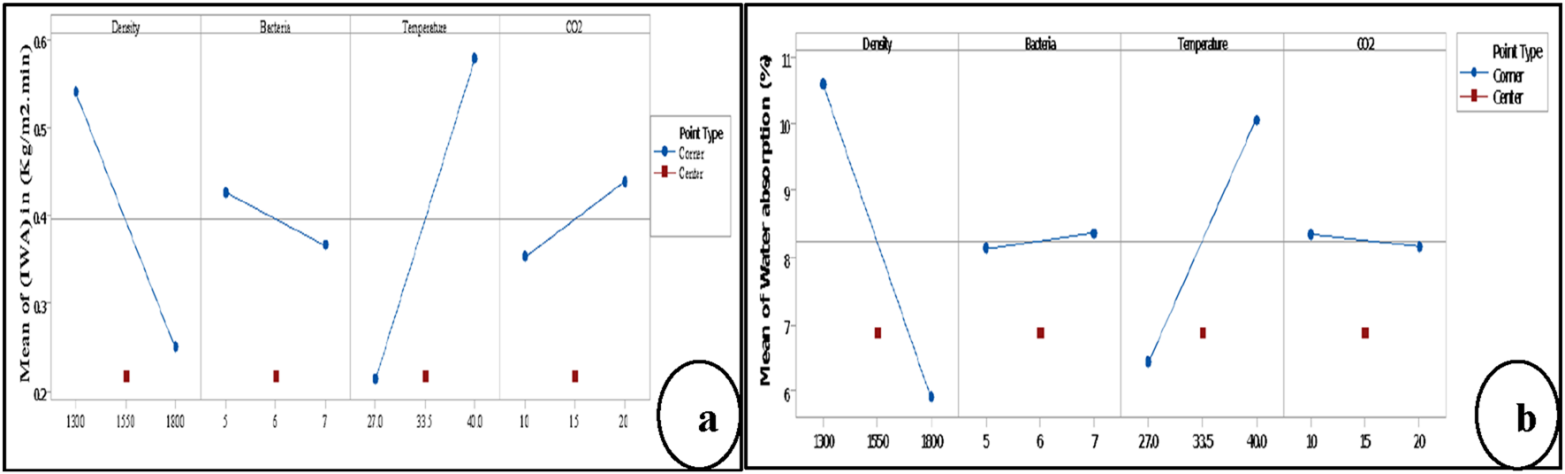
The main effects plots of the factors by factorial analysis (**a**) response is initial water absorption (**b**) response is water absorption.

The results presented in Fig. 7 demonstrate the performance of each factor for both responses. In Fig. 7a, the increase of density of B-FCB and *B. tequilensis* were caused by decrement on IWA, while the increase of temperature and CO_2_ levels were caused by increment on IWA. Therefore, the convenient conditions for the self-healing of B-FCB surface may occur at high density levels and *B. tequilensis* when the temperature and CO_2_ are at low levels.

The same conditions of density and temperature were also suggested by the WA results, as shown in Fig. 7b. However, the performance of CO_2_ and *B. tequilensis* in the results of WA were contrary to the performance of IWA. This finding may be interpreted as follows: at the surface of B-FCB, *B. tequilensis* enzymes react directly with CO_2_ atoms available in the atmosphere of the chamber during curing time. Consequently, *B. tequilensis* with high concentration was preferred to react directly with CO_2_ at a low level, decreasing IWA. Due to the difficulties of penetration of CO_2_ atoms inside the B-FCB, a high level of CO_2_ was required during curing time in the chamber to react with *B. tequilensis* enzymes resulting in the decrement of WA.

**Figure 8:**
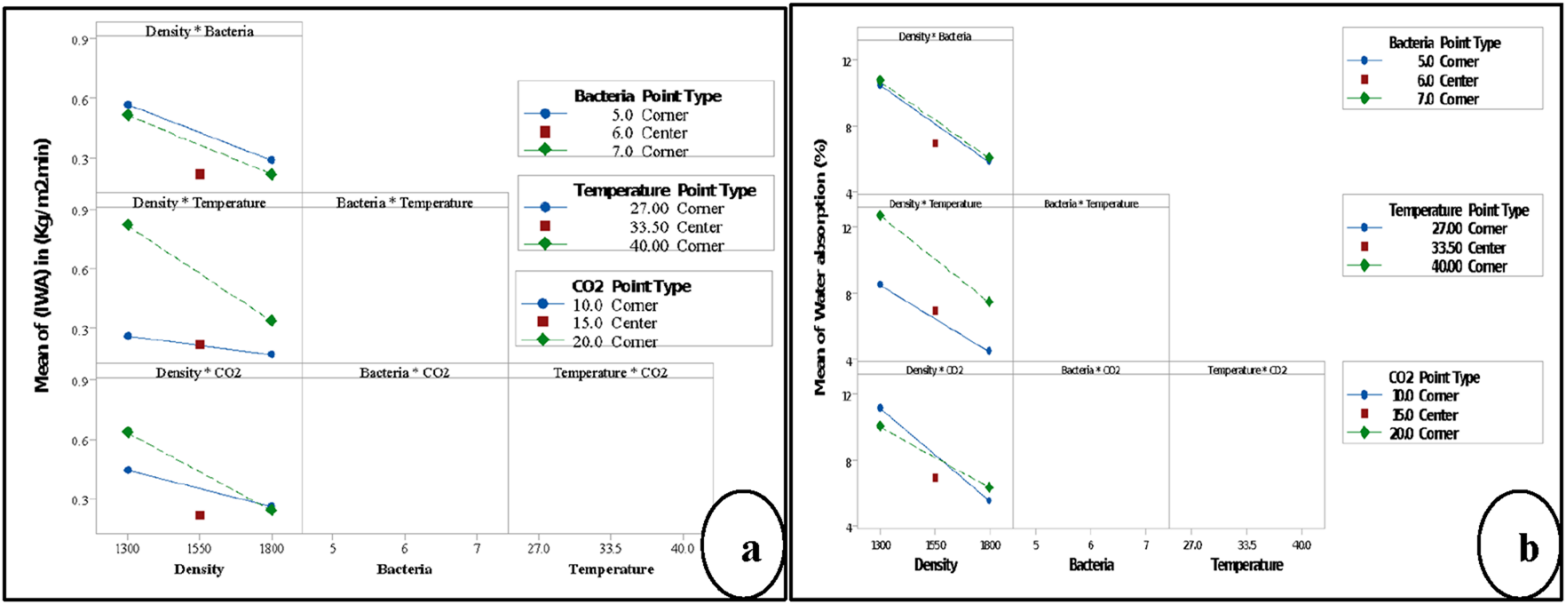
Interaction plot of the factors by factorial analysis (**a**) response is initial water absorption (**b**) response is water absorption.

Moreover, the interaction between density*CO_2_ of IWA and WA was the highest compared to other interactions, as shown in Fig. 8a,b. The interaction results confirmed the performance of the single factors of each response. However, there is an important point that must be highlighted in this section, the change occurred in the performance of the main factors and their interactions are normal because water absorption used to measure the quantity of water that penetrates into B-FCB for 24 h, and one minute was used to monitor water penetration by initial water absorption test. Therefore, when the water exceeds the surface of B-FCB, it can face other factors.

According to the previous discussion, it can be summarised that the performance of the main factors changes from one response to another. In addition, the results cannot rely on the performance of the main factors only. The interactions between factors must be considered before the optimisation process.

### Response surface methodology (RSM)

#### The normality and accuracy of the design

**Figure 9:**
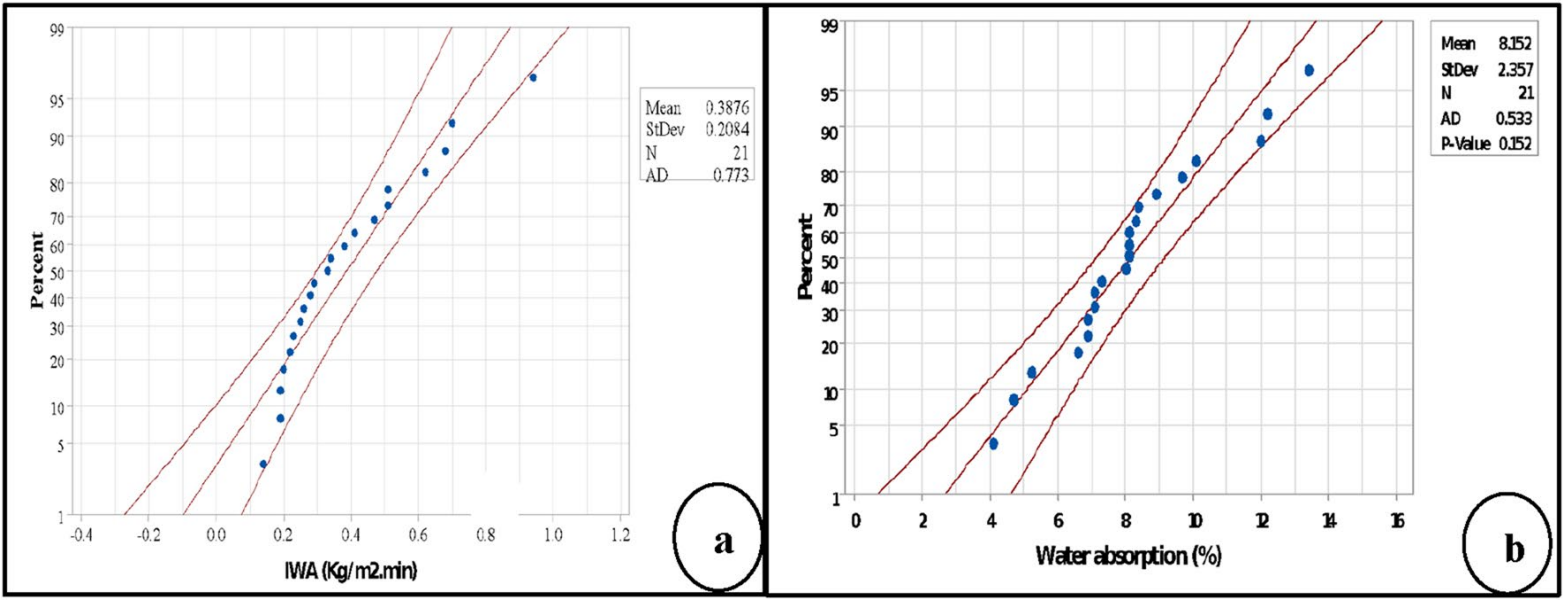
Accuracy of the design using probability plots by RSM analysis (**a**) initial water absorption of B-FCB (**b**) water absorption of B-FCB.

In this research, RSM used extended design because the curvature was significant in the factorial analysis. Therefore, 8 axial runs and 2 centre runs to factorial design. The results of probability plots used for both responses verified the normality of the work. The probability plot in statistical analysis can determine if the distribution curve is normal or not, which mainly depends on the P-value^31^. When the P-value < 0.05, that means the data distribution is non-normal, and the confidence level of the data is not 95% or above. In contrast, for the P-value ≥ 0.05, the distribution curve is assumed normal, and the accuracy of the results is high 95% and above. The results demonstrated that the P-value in probability plots of IWA and WA were 0.773 and 0.152, respectively, which satisfies the condition for normal distribution because the P-values > 0.05 of both responses. Therefore, the blue dots representing the runs did not cross the boundaries red lines as shown in Fig. 9a,b, except one dot of IWA was out on the red boundary line. According to P-values, the level of accuracy of IWA was higher than WA. This finding corroborates that the error of the results of both responses is below 5%, which reflect a high level of accuracy of the model.

#### The significant factors to the responses in RSM

**Figure 10:**
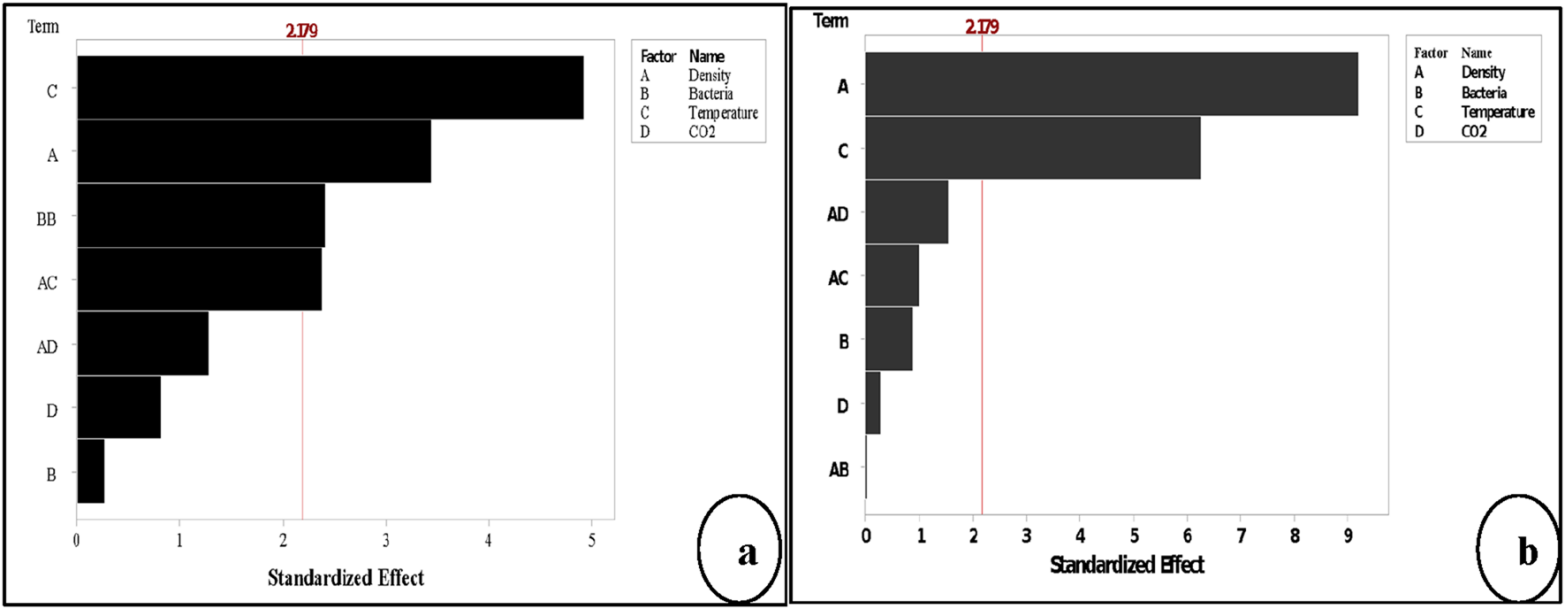
Pareto chart of the standardised effects by RSM analysis α = 0.05 (**a**) response is initial water absorption (**b**) response is water absorption.

The performance of the factors and their interactions in RSM analysis slightly change compared to factorial analysis. According to ANOVA results shown in Table 3, the remaining factors as significant from factorial analysis up to RSM for both responses were density and temperature with P < 0.05. However, *B. tequilensis* and CO_2_ were significant in factorial analysis of IWA results, which were insignificant when design extended to RSM. The interest of this finding, *B. tequilensis* as a single factor, was insignificant in IWA, but it was significant as individual interaction (square) as shown in Fig. 10a. The *B. tequilensis* itself was significant and positively affected the response variation, but it does not interact with other factors at this stage of analysis.

**Table 3:** ANOVA of RSM analysis for the effect of factors and their interactions to the responses.

Source	P-value (IWA)	Effect (IWA)	P-value (WA)	Effect (WA)
Model	0.002	–	0.000	–
Blocks	0.018	–	0.168	–
Linear	0.001	–	0.000	–
Density	0.005	− 0.200	0.000	− 2.570
Bacteria	0.789	− 0.000	0.388	0.250
Temperature	0.000	3.300	0.000	1.750
CO_2_	0.428	− 2.700	0.779	− 0.080
Square	0.033	–	–	–
Bacteria*bacteria	0.033	0.143	–	–
2-way interactions	0.057	–	0.337	–
Density*bacteria	–	–	0.969	− 0.012
Density*temperature	0.462	0.750	0.337	− 0.312
CO_2_*density	0.224	− 0.750	0.114	0.488
Lack-of-fit	0.001	–	0.015	–

The results support the original hypothesis of this research that the course of events is as follows: the reaction of the bacterial enzymes with the natural carbonation in the concrete may help to improve the self-healing process by accelerating the CO_2_ sequestration in concrete pores in the form of CaCO_3_^32^. The WA results confirmed this hypothesis, where the main significant factors were density and temperature, while CO_2_ was insignificant because it took a long time to penetrate deeply into the concrete^33^. Therefore, *B. tequilensis* performance in water absorption was lower than in initial water absorption and the healing process due to the low concentration of CO_2_ inside the B-FCB, especially when CO_2_ is used in bio-concrete as an alternative for urea as in this research^34^.

The interactions of density*temperature in IWA was significant, while other interactions for both responses were insignificant, as shown in Fig. 10a, b. Moreover, interactions such as density*CO_2_ for both responses was significant during factorials analysis and became insignificant in RSM design.

#### Effect of *B. tequilensis* to the responses with each factor

**Figure 11:**
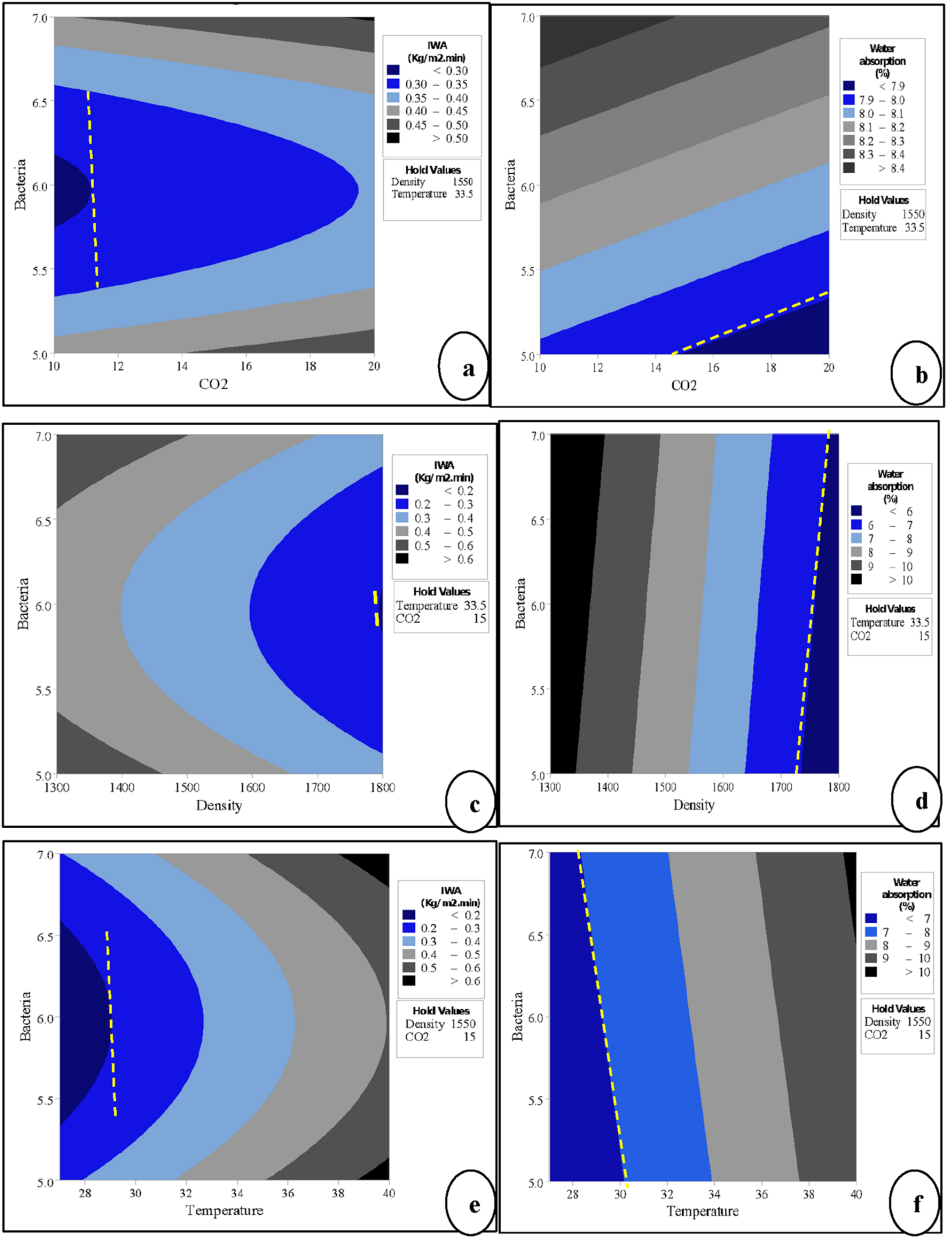
Counter plots of the responses with different factors (**a**,**c**,**e**) counter plots for IWA and (**b**,**d**,**f**) counter plots for WA.

A useful way to evaluate the performance of any system is to study the impact of the inputs on the outputs. The previous sections discuss the performance of individual and interaction influences self-healing of B-FCB, indicated via IWA and WA. Contour plots were constructed for all the cases for this purpose, as shown in Fig. 11. The yellow line was added to each contour plot to highlight the low water level absorbed by B-FCB. The relation between *B. tequilensis* and CO_2_ for IWA and WA are presented in Fig. 11a,b, respectively. It can be realised that in contour (a), the centre level of *B. tequilensis* is the best level to reduce IWA when the CO_2_ is at a low level. Meanwhile, the lower WA may occur when the *B. tequilensis* at a low level while CO_2_ at the centre or high level. This is because Ca that is available in cement was firstly leached out from calcium silicate minerals to form the Ca-depleted C–S–H zone on the surface of B-FCB, which in the role was precipitated as CaCO_3_ locally around the surface of the particle under the accelerated CO_2_ via carbonation process^35^. The healing occurred on the surface of B-FCB pores before the pores inside the B-FCB, which obstructs the penetration of CO_2_ into B-FCB pores. Consequently, the high level of CO_2_ resulted in the highest self-healing and the highest decrement of WA.

The relation between *B. tequilensis* and density was stronger with IWA than WA, as shown in contour plots Fig. 11c,d. At a high density and centre level of *B. tequilensis,* the highest decrement of IWA. The same performance of both factors on WA was performed in terms of density. However, the *B. tequilensis* was performed positively in all levels low, centre and high.

Figure 11e,f show the strong relation between temperature and *B. tequilensis* has appeared to be distinct in IWA, especially when *B. tequilensis* at the centre level and temperature at a low level. On the other hand, the lower penetration of WA takes place into B-FCB when the temperature blows 30 °C and *B. tequilensis* at all levels.

The fluctuated performance that appeared on contour plots between the two responses comes out with the following finding shows that the effect of the factors on the surface and inside of B-FCB is not the same. Therefore, the optimisation of both responses must be analysed and come out with a statistical model of each response.

#### Optimisation of minimum IWA and WA in B-FCB

**Figure 12:**
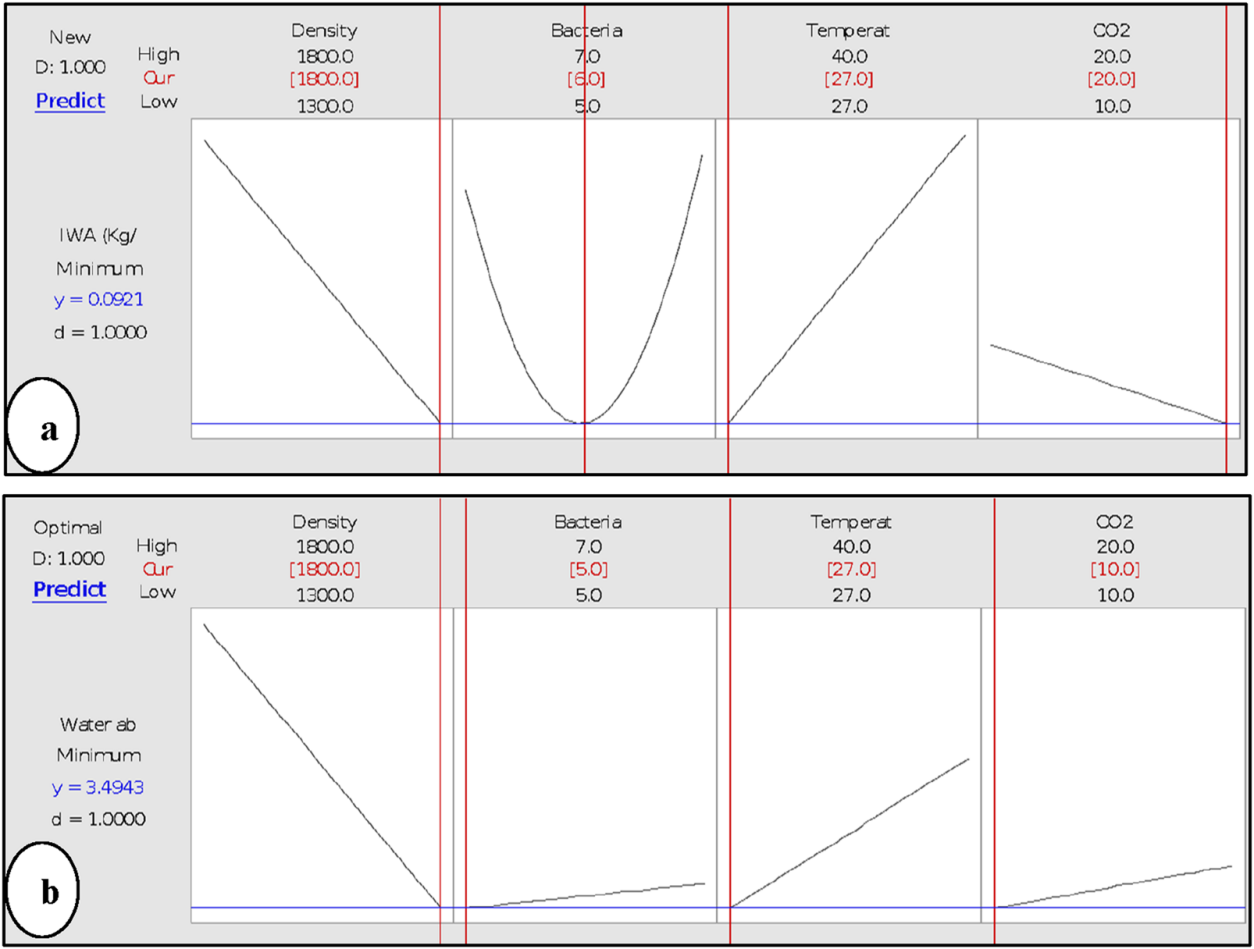
Optimisation plot for minimum (**a**) initial water absorption (**b**) water absorption of B-FCB.

The optimisation plot of the minimum IWA and WA are shown in Fig. 12. The solid red lines indicate the setting of each factor that led to the lowest attainment of responses. Meanwhile, the dotted blue lines represent the predicted IWA and WA on B-FCB.

The results of IWA predicted the best level of each factor by RSM analysis to produce B-FCB with a surface that has high resistance to water. The minimum predicted IWA was 0.0921 (kg/m^2^.min) at the following levels: 1800 kg/m^3^, 3 × 10^6^ cell/mL, 27 °C and 20% of density, *B. tequilensis*, temperature and CO_2_, respectively as shown in Fig. 12a. On the other hand, the minimum percentage of WA was 3.4943 at 1800 kg/m^3^, 3 × 10^5^ cell/mL, 27 °C and 10% of density, *B. tequilensis*, temperature and CO_2_ respectively, as shown in Fig. 12b. The difference of predicted levels of the factors in both responses occurred at CO_2_ and *B. tequilensis* concentrations, while the density and temperature levels were the same. This finding confirmed the previous discussion in factorial and RSM results, which reflect the strong relationship between CO_2_ sequestration and *B. tequilensis,* especially at the surface of B-FCB. Therefore, the minimum IWA requires a high level of CO_2_ and *B. tequilensis,* while the minimum WA can be achieved at low levels of the same factors. The empirical models of both responses were developed via RSM analysis after the IWA and WA of B-FCB. The model derived from the ANOVA results explains the concrete relationship between the independent variables (significant terms) and both responses. The final regression equations in Uncoded Units of initial water absorption and water absorption and are given in Eqs. (3) and (4) respectively.

3 $$\begin{aligned} {\text{IWA }}\left( {{\text{kg}}/{{\text{m}}^2}{\text{min}}} \right) & = 1.10 \, + \, 0.002136{\text{ Density }} - \, 1.713{\text{ Bacteria }} + \, 0.1207{\text{ Temperature }} \\ & \quad + \, 0.0711{\text{ C}}{{\text{O}}_2} + \, 0.1436{\text{ Bacteria }}*{\text{ Bacteria }} - \, 0.000060{\text{ Density }}*{\text{ Temperature }} - \, 0.000042{\text{ Density }}* \, {\text{ C}}{{\text{O}}_2} \\ \end{aligned} $$

4 $$\begin{aligned} {\text{Water absorption }}\left( \% \right) & = 12.4 \, - \, 0.0094{\text{ Density }} + \, 0.33{\text{ Bacteria }} + \, 0.567{\text{ Temperature }} \\ & \quad - \, 0.621{\text{ C}}{{\text{O}}_2} - \, 0.00005{\text{ Density }}*{\text{ Bacteria }} \\ & \quad - \, 0.000192{\text{ Density }}*{\text{ Temperature }} + \, 0.000390{\text{ Density }}*{\text{ C}}{{\text{O}}_2} \\ \end{aligned} $$

### Microstructure analysis

#### SEM study of FCB and B-FCB

**Figure 13:**
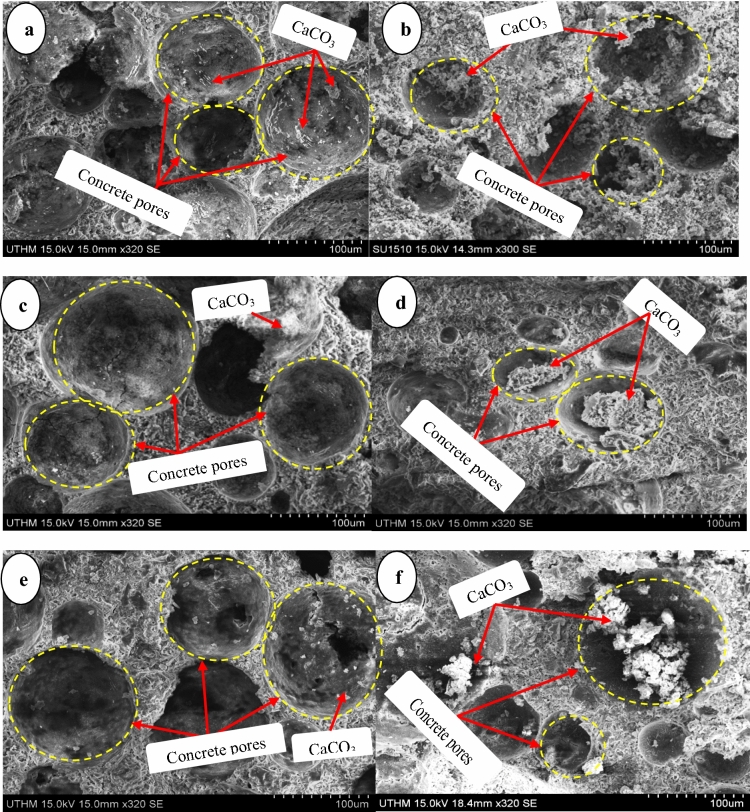
Comparison of SEM images between (**a**) FCB under 1300 kg/m^3^ (**b**) B-FCB with 1300 kg/m^3^ (**c**) FCB with 1550 kg/m^3^ (**d**) B-FCB with 1550 kg/m^3^ (**e**) FCB with 1800 kg/m^3^ (**f**) B-FCB with 1800 kg/m^3^.

The healing of B-FCB pores compared to FCB are demonstrated in Fig. 13. There are calcifications of CaCO_3_ in the pores of B-FCB under different levels of densities 1300 kg/m^3^, 1550 kg/m^3^, 1800 kg/m^3^ as shown in Fig. 13b,d, ), respectively. However, the FCB specimens Fig. 13a–c, have a little calcification of CaCO_3_ due to natural carbonation reaction between CO_2_ calcium-silicate-hydrates (C–S–H) and portlandite Ca(OH)_2_^36^. Consequently, the healing process in B-FCB occurred due to the availability of *Bacillus tequilensis*^32^. *Bacillus tequilensis* has the ability to produce CA and urease enzymes which are main players on CaCO_3_ formation in B-FCB^37^. Nevertheless, the role of curing condition and density of concrete cannot be neglected because density, CO_2_ concentration, and temperature are all factors that mainly affect the carbonation process. However, the natural carbonation is very slow in terms of CaCO_3_ formation; therefore, the self-healing in the specimens of FCB was very low, too, as demonstrated in Fig. 13a,c,e^38^.

#### Elements of FCB and B-FCB

**Figure 14:**
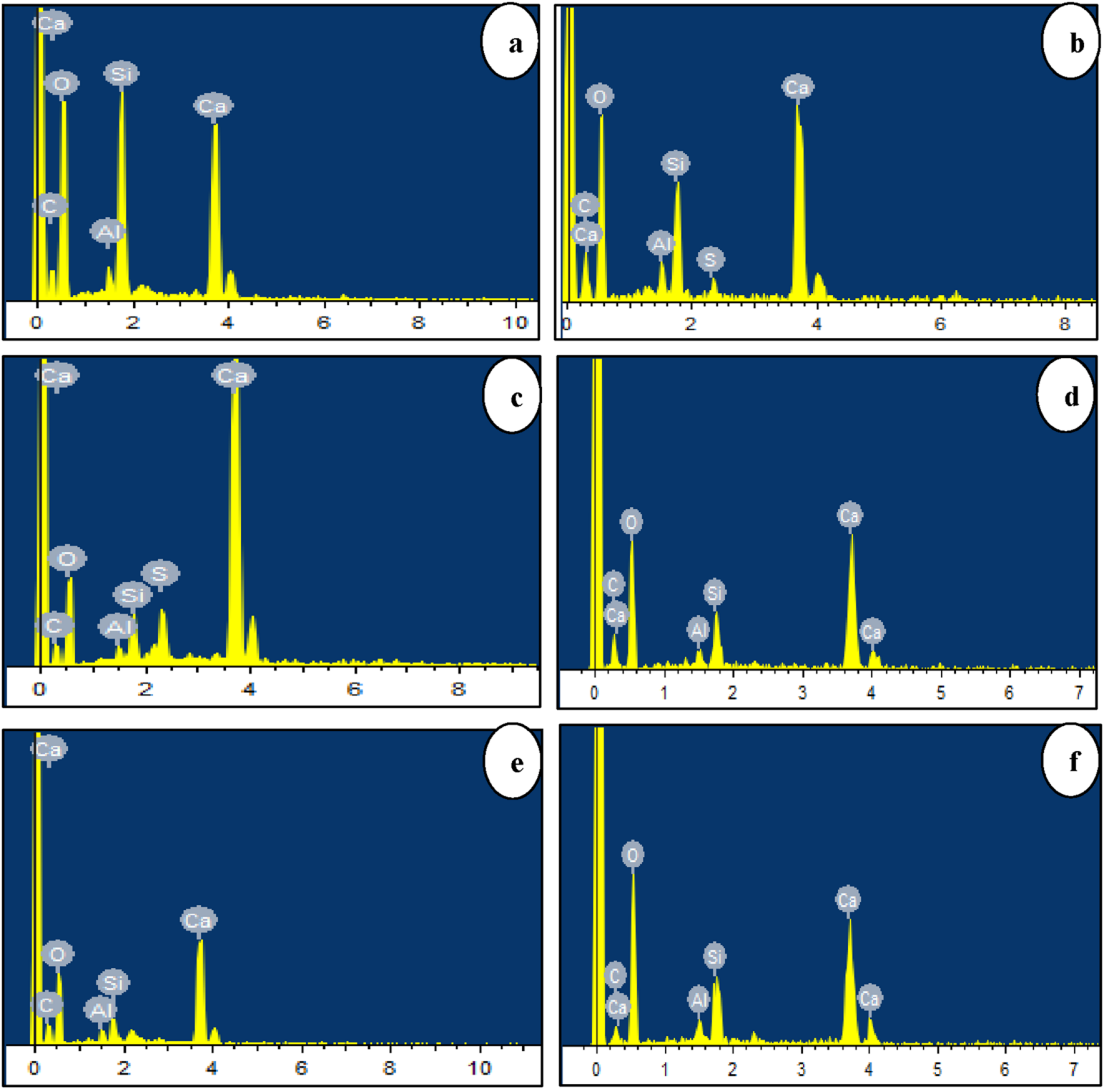
EDX images compared between (**a**) FCB with 1300 kg/m^3^, (**b**) B-FCB with 1300 kg/m^3^, (**c**) FCB with 1550 kg/m^3^, (**d**) B-FCB with 1550 kg/m^3^, (**e**) FCB with 1800 kg/m^3^, and (**f**) B-FCB with 1800 kg/m^3^.

Three elements were monitored by EDX spectrum results, namely Ca, C, and O, which are the elements responsible for composite CaCO_3_^39^. The average weight of the three points in each sample was used to investigate the change of weight in each element of FCB and B-FCB samples. The results indicated that the weight of Ca, C, and O changed from one run to another, which confirmed the significant effect of the factors used on the self-healing process, as shown in Fig. 14. However, it was released that the weight of the Ca in B-FCB specimens was lower than FCB samples and the weight of C and O were higher in B-FCB compared to FCB at the same run as shown in Table 4. The change of the weight of the elements in FCB and B-FCB under the same run was due to the role of *B. tequilensis* and their enzyme reactions, which used the available Ca in cement to precipitate CaCO_3,_ resulting in self-healing of B-FCB pores. Alongside this finding, the CO_2_ concentration and temperature strongly affect the percentage of the weight of each element from one run to another. Also, density plays a vigorous role in the weight of elements. The specimens with high levels of densities have high amounts of cement, which contain high percentages of Ca. EDX results can come out with this finding: self-healing via CaCO_3_ mainly affected by *B. tequilensis* concentration, concrete density, and curing conditions.

**Table 4:** The chemical weight (%) of chemical elements C, O, and Ca in FCB and B-FCB.

Run no	Weight (%) of FCB elements			Weight (%) of B-FCB elements		
	C	O	Ca	C	O	Ca
1	9.4	45.9	30.9	12.6	59.86	25.2
2	20.7	27.8	49.0	25.0	40.6	28.6
3	9.7	53.5	25.4	10.6	52.9	26.2
4	8.5	24.1	63.3	24.1	41.1	29.2
5	4.2	32.5	60.6	5.9	53.3	32.4
6	4.94	32.6	56.6	13.0	51.2	27.0
7	18.9	17.4	61.3	37.3	33.6	23.75
8	4.6	42.8	40.8	7.0	51.8	32.7
9	5.2	40.1	53.2	16.5	39.1	35.2
10	5.1	37.0	55.2	8.0	46.3	37.7
11	6.6	41.8	47.2	10.7	55.1	25.7
12	7.2	47.6	40.5	9.4	52.3	36.1
13	10.4	53.2	30.0	11.5	57.6	23.8
14	6.9	43.2	42.6	8.9	50.9	33.0
15	5.2	43.8	53.1	7.0	46.3	45.3
16	5.3	44.6	50.4	5.9	48.6	41.7
17	7.2	39.2	47.7	8.4	42.3	32.5
18	6.2	44.6	54.3	8.8	50.9	33.03
19	4.3	47.8	43.1	5.2	55.7	38.6
20	6.7	41.8	45.6	7.8	57.9	29.4
21	6.4	39.8	51.3	8.9	48.7	33.8

#### Crystallinity analysis of FCB and B-FCB

**Figure 15:**
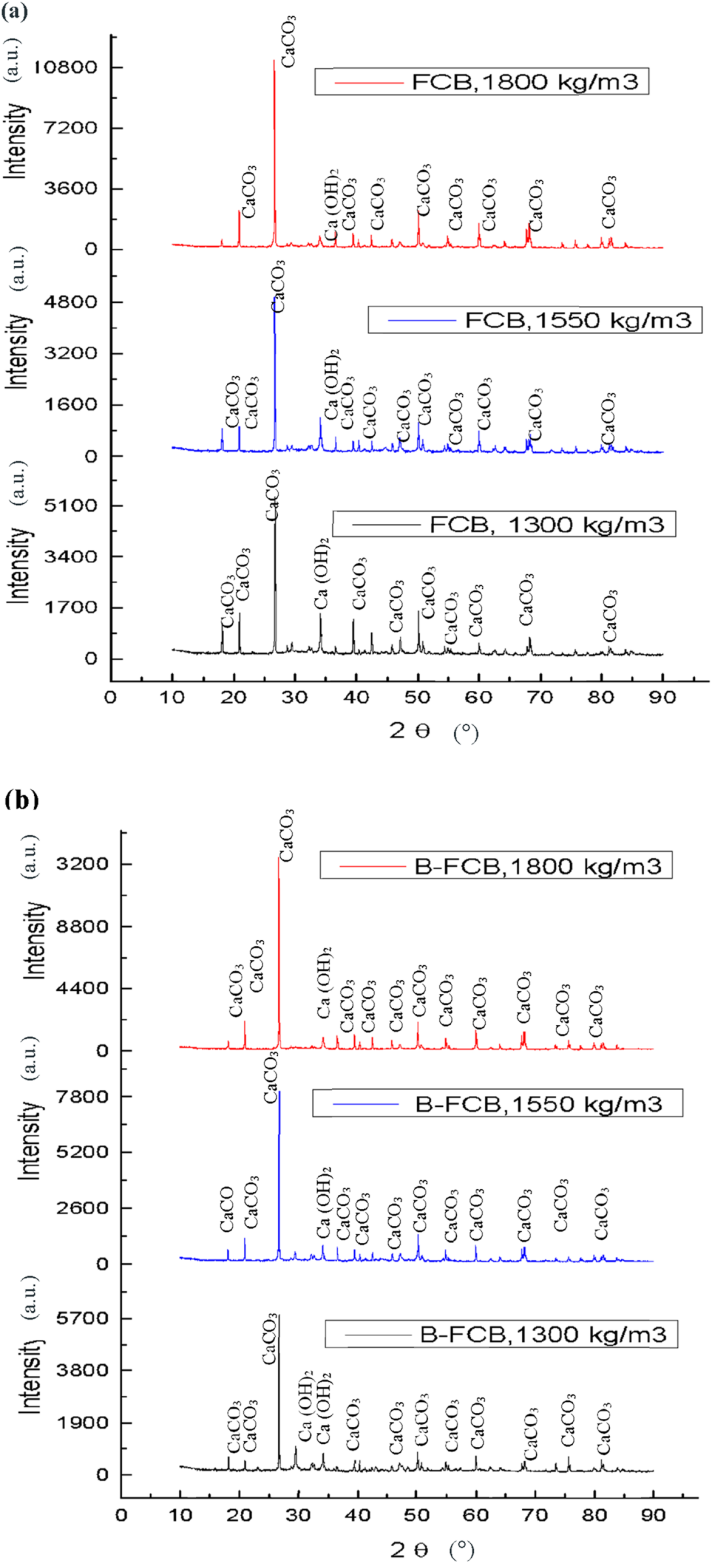
XRD spectra of produced precipitate CaCO_3_ with different level of densities (**a**) FCB specimens (**b**) B-FCB specimens at 28 days.

XRD results demonstrated the intensity of calcite precipitation, especially CaCO_3,_ at different densities and curing conditions, as presented in Fig. 15. The intensity of calcite precipitation in FCB and B-FCB specimens increase when the level of density increases. This finding reflects the normal relation between Ca and CaCO_3_ precipitations, whereas, at high levels of density, the amount of cement is high compared to low density and the amount of Ca. However, the intensity was higher in the specimens containing *B. tequilensis* at the same level of density and curing conditions, which approved the functional role of *B. tequilensis* on self-healing and precipitation of CaCO_3_. Also, the number of peaks is different from one run to another, which may change the percentage of crystallinity precipitation. Therefore, the percentage of crystallinity was calculated using Eq. (5), as shown in Table 5^40^. The results proved that the crystallinity of most samples containing *B. tequilensis* are higher than the control samples. The increase of crystallinity in B-FCB was due to the reaction of *B. tequilensis* enzymes and CO_2_ along the curing time in the chamber resulting in an increment of CaCO_3_ amount^41^. The difference in crystallinity from one run to another reflects the strong relationship between the factors used and CaCO_3_ formation, especially the CO_2_ and *B. tequilensis* concentrations.

5 $${\text{Crystallinity\;}}\left( {\text{\% }} \right) = \frac{{{\text{area\;of\;each\;peaks}}}}{{{\text{Total\;area\;of\;all\;peaks}}}}{\text{\;}} \times {\text{\;}}100$$

**Table 5:** Crystallinity percentage of FCB and B-FCB.

Run no	Crystallinity of (FCB) specimens			Crystallinity of (B-FCB) specimens		
	Area of peaks	Total area	Crystallinity (%)	Area of peaks	Total area	Crystallinity (%)
1	681.9	1047.44	65.1	725.2	1062.9	68.2
2	6689.4	9876.4	67.7	7000.0	9678.2	72.3
3	630.20	1168.71	53.9	723.4	1230	59.0
4	5852.2	7724.8	75.7	6577.4	8060.1	81.6
5	6706.0	9856.3	67.9	7425.7	10,361.1	71.6
6	6372.6	8014.6	79.5	5986	7754.6	77.2
7	6941.2	8613.9	80.5	6573.8	7960.0	82.5
8	6543.0	9083.8	71.9	6964.4	9662.0	72.1
9	747.7	1277.9	58.5	866.3	1348.8	64.2
10	772.7	1308.2	59.0	727.4	1185.5	61.3
11	690.3	1219.4	56.6	758.1	1260.3	60.1
12	5734.8	8301.9	69.0	5816.7	8430.2	69.9
13	5888.5	7650.2	76.9	6922.4	9008.4	76.8
14	4439.1	8370.1	53.0	6817.0	9161.9	63.5
15	7812.4	11,266.8	69.48	8017.4	10,260.3	78.14
16	6820.7	9060.9	75.2	7377.6	9062.5	81.4
17	7381.2	8758.7	84.2	7511.0	8974.7	83.7
18	3670.2	5844.3	62.8	4145.0	5900.8	70.2
19	7322.9	9877.6	74.1	7750.0	10,497.0	73.8
20	6686.4	8915.8	74.9	6529.2	8225.6	79.3
21	6445	8481.9	76.2	6532.4	8402.4	77.7

## Conclusion

Each response clearly displayed the healing in B-FCB, which confirmed the significant role of the factors and their interactions to the results. The IWA was indicated that the heal appeared on the surface of B-FCB while the WA healing occurred on the whole specimens.

The best conditions for *B. tequilensis* to cause self-healing on the surface of B-FCB could be 1800 kg/m^3^, 3 × 10^6^ cell/mL, 27 °C, and 20% of CO_2,_ which resulted in the minimum IWA 0.0921 kg/m^2^.min. While, under the following condition, 1800 kg/m^3^, 3 × 10^5^ cell/mL, 27 °C and 10% of CO_2_ was suggested to cause the optimal healing of the pores inside B-FCB, resulting in a minimum ratio of WA 3.4943%. The role of each factor and their interactions on both factorial and RSM as optimised methods were to raise up the performance of *B. tequilensis* and healing ratio of B-FCB pores.
